# An Accelerated Failure Time Model to Predict Cause-Specific Survival and Prognostic Factors of Lung and Bronchus Cancer Patients with at Least Bone or Brain Metastases: Development and Internal Validation Using a SEER-Based Study

**DOI:** 10.3390/cancers16030668

**Published:** 2024-02-04

**Authors:** Phillip Oluwatobi Awodutire, Michael W. Kattan, Oluwatosin Stephen Ilori, Oluwatosin Ruth Ilori

**Affiliations:** 1 Cleveland Clinic, Cleveland, OH 44195, USA; kattanm@ccf.org; 2 Ladoke Akintola University of Technology Teaching Hospital, Ogbomosho 212102, Nigeria; osilori@lautech.edu.ng (O.S.I.); orilori@lautech.edu.ng (O.R.I.)

**Keywords:** cause-specific survival, accelerated failure time model, Zografos–Balakrishnan log-normal distribution, lung cancer, prognostic

## Abstract

**Simple Summary:**

The study introduces a ZBLN-based AFT model for predicting 3-year and 5-year survival in CSLCD patients with metastases, showing superior performance over other models. Proper selection of survival analysis models, like ZBLN, is crucial for accurate predictions in clinical settings. Comparison of survival outcomes indicates higher rates for lung cancer patients with bone metastases alone compared to those with bone and brain metastases. Associations between gender, race, treatment modalities, and histological types are noted.

**Abstract:**

Background: This study addresses the significant challenge of low survival rates in patients with cause-specific lung cancer accompanied by bone or brain metastases. Recognizing the critical need for an effective predictive model, the research aims to establish survival prediction models using both parametric and non-parametric approaches. Methods: Clinical data from lung cancer patients with at least one bone or brain metastasis between 2000 and 2020 from the SEER database were utilized. Four models were constructed: Cox proportional hazard, Weibull accelerated failure time (AFT), log-normal AFT, and Zografos–Balakrishnan log-normal (ZBLN). Independent prognostic factors for cause-specific survival were identified, and model fit was evaluated using Akaike’s and Bayesian information criteria. Internal validation assessed predictive accuracy and discriminability through the Harriel Concordance Index (C-index) and calibration plots. Results: A total of 20,412 patients were included, with 14,290 (70%) as the training cohort and 6122 (30%) validation. Independent prognostic factors selected for the study were age, race, sex, primary tumor site, disease grade, total malignant tumor in situ, metastases, treatment modality, and histology. Among the accelerated failure time (AFT) models considered, the ZBLN distribution exhibited the most robust model fit for the 3- and 5-year survival, as evidenced by the lowest values of Akaike’s information criterion of 6322 and 79,396, and the Bayesian information criterion of 63,495 and 79,396, respectively. This outperformed other AFT and Cox models (AIC = [156,891, 211,125]; BIC = [158,848, 211,287]). Regarding predictive accuracy, the ZBLN AFT model achieved the highest concordance C-index (0.682, 0.667), a better performance than the Cox model (0.669, 0.643). The calibration curves of the ZBLN AFT model demonstrated a high degree of concordance between actual and predicted values. All variables considered in this study demonstrated significance at the 0.05 level for the ZBLN AFT model. However, differences emerged in the significant variations in survival times between subgroups. The study revealed that patients with only bone metastases have a higher chance of survival compared to only brain and those with bone and brain metastases. Conclusions: The study highlights the underutilized but accurate nature of the accelerated failure time model in predicting lung cancer survival and identifying prognostic factors. These findings have implications for individualized clinical decisions, indicating the potential for screening and professional care of lung cancer patients with at least one bone or brain metastasis in the future.

## 1. Introduction

Lung cancer has been considered one of the leading causes of cancer deaths worldwide, accounting for 1.38 million deaths yearly (18.2% of all cancer deaths). According to the latest GLOBOCAN estimates, 2,094,000 new cases of lung cancer were diagnosed globally in 2018, making lung cancer the leading cancer incidence worldwide. With an estimated 1,369,000 cases, lung cancer is the second most common cancer in men after prostate cancer and the second most common cancer in women after breast cancer, with 725,000 cases. The cumulative lifetime risk of age-standardized lung cancer diagnosis is 3.8% among men and 1.77% among women [1,2]. Lung cancer also has the highest mortality rate among all types of cancer. It is responsible for more deaths than breast, colorectal, and prostate cancers combined. Lung and bronchus cancer are responsible for the most deaths, with 127,070 people expected to die from this disease [3]. Lung cancer diagnosis and survival are challenging, since it is also one of the most frequently diagnosed cancers [4]. Lung cancer can spread from the lungs to other parts of the body through the bloodstream or the lymphatic system, a process known as metastasis. Bone is the most frequent target site of distant metastasis for lung cancer, affecting up to 14–40% of patients, yet its clinical features have not been clearly described [5,6,7,8]. Bone metastasis can have devastating consequences for lung cancer patients, as it can lead to severe pain, pathological fractures, spinal cord compression, hypercalcemia, and reduced mobility. These complications, also known as skeletal-related events (SREs), can impair the quality of life and survival of patients, as well as increase health care costs and burden. The prognosis of lung cancer patients with bone metastasis is poor, with a reported average survival time of six to 10 months after metastasis. The 5-year survival rate for these patients is less than 5%, compared to 15% for lung cancer patients without bone metastasis. The median survival time for lung cancer patients with bone metastasis is about three months, which is similar to that of patients with brain metastasis, another common and fatal complication of lung cancer. When treating lung cancer with metastases, it is essential to know the prognostic factors and prognosis after bone or brain metastasis, as they are the commonest ones. Many studies have used survival models to explore the survival times of lung cancer patients in different medical scenarios, focusing on the Kaplan–Meier and Cox proportional- hazard model approaches. Alomaish and his colleagues investigated whether interstitial lung disease affects the survival times of patients with lung cancer [9]. Another study used the Cox model to explore hyaluronan to predict metastasis and survival rates in patients with small-cell lung cancer [10]. Meng et al. investigated prognostic factors in advanced lung adenocarcinoma with one to five bone-only metastases and developed a nomogram model to estimate overall survival in the patient [11]. Many patients with lung cancer report being late for treatment, with the tumor in advanced stages of development. The results show that the 5-year survival rate for lung cancer patients is between 10% and 20%, as reported by Stanley [12] and Freise et al. [13], indicating a poor prognosis. Wang et al. developed and validated a predictive model to predict survival for lung adenocarcinoma. In their work, they developed a model that predicts one, three, and five years of overall survival for lung cancer patients [14]. There is a paucity of data on the combination of the three metastases considered in this current study—bone alone, brain alone, and bone and brain—and their relationship to survival time. Also, there is a shortage of studies on predictors of lung cancer patients who experience death due to lung cancer. This is, in other words, novel research that will go a long way towards informing clinicians about the management of lung metastases among patients.

In this review, the Cox proportional hazard model is the model most prominently and widely used to model the survival of cancer patients, including lung cancer. However, the Cox model depends on the proportional hazard assumption and may not be appropriate if it is violated. Parametric survival models presume that the survival time follows a particular probability distribution, such as the exponential, Weibull, or log-normal distribution. These models provide insight into the fundamental survival mechanism by estimating the parameters of the selected distribution. When the assumption of proportional hazard is not met, or when the hazard function varies over time, they are useful as an alternative. Moreover, parametric models give more consistent and efficient estimates than the Cox model. Model validation techniques, such as cross-validation and goodness-of-fit evaluations, can assist in evaluating the performance of various models and in guiding the selection procedure. Although the parametric approach has been used in some cancer studies [15,16,17], this method has not been considered for lung cancer studies. The development of more sophisticated survival analysis techniques, such as advanced probability distributions, which differ from the commonly used probability distributions for the accelerated failure time model, is attributable to advances in statistical methods and computational capacity. These methods add to our understanding of complex survival scenarios and facilitate high-performing predictive models. The Zografos–Balakrishnan log-normal distribution (ZBLN), a generalized form of the log-normal distribution, is explored as a baseline distribution for the accelerated failure time model to achieve this aim. The ZBLN distribution was considered because of its flexibility in handling skewed data. This model has explored this distribution for breast cancer studies [18,19]. This study aimed to develop and validate a prediction model for 3- and 5-year cause-specific survival of lung cancer with at least one bone or brain metastasis (CSLCD) and to investigate the influence of some prognostic factors. We sought to identify and compare the derived model for fit, discrimination, calibration, and clinical utility compared to other prediction models. We further aimed to compare the survival times among the categories of metastases (those with bone-only, brain-only, and a combination of the two) using the acceleration factor. A dataset of 20,412 patients with lung cancer with bone and brain metastases from 17 registries between 2000 and 2020 from the SEER database was used to develop and validate this model.

## 2. Materials and Methods

### 2.1. Patients Included in the Study

For this population-based study, the updated Surveillance, Epidemiology, and End Results (SEER) records for 17 custom data registries, supplemented with additional treatment fields, served as the primary data source. SEER*Stat Software version 8.4.1.2 (https://seer.cancer.gov/seerstat/ (accessed on 30 October 2023)) was used to extract the information. The SEER program, maintained by the National Cancer Institute, is recognized as the largest publicly available cancer dataset worldwide. It encompasses 17 population-based cancer registries and covers approximately 26% of the population of the United States, spanning multiple geographic regions [3]. Patients diagnosed with lung cancer as their primary cancer between 2000 and 2020, with bone metastases, brain metastases, or bone and brain metastases, were enrolled in the research. The classification of tumors was based on their primary presentation site, using the International Classification of Diseases for Oncology, Third Edition (ICD-O-3). Patients with unknown and other metastatic sites were excluded. Furthermore, patients less than 20 years of age were excluded from the study because of the small sample within the group. Since the SEER database is publicly accessible, obtaining informed consent from the patients for this study was unnecessary, and it was considered exempt from review by the Cleveland Clinic Ethics Committee.

### 2.2. Demographic and Clinical Variables

Information extracted from the SEER database is described in this section with their model code. The inclusion criteria were as follows: the site-recoded ICD-O-3 2023 Revision Expanded criterion was “Lung and Bronchus”; the SEER cause-specific death classification was “Dead (attribute to this cancer dx)”; diagnosed between 2000 and 2020. The variables extracted were the age of patients at presentation, gender, histological tumor grade, histology classification, treatment modality, the total number of in situ malignant tumors, combination of metastases, race, and primary tumor sites. Gender was classified as Male (1) and Female (2). Race was categorized as American Indian (1), Asian (2), Black (3), and White (4). The primary sites of the tumor were the main bronchus (1), upper lobe (2), lower lobe (3), lungs NOS (4), and overlapping lesion of the lung (5). Treatment modalities were aggregated into four groups, namely: no intervention (1), chemotherapy only (monotherapy) (2), radiation + surgery (bimodal therapy) (3), and radiation + surgery + chemotherapy (trimodal therapy) (4). Histological grades were classified as follows: well-differentiated (1), moderately differentiated (2), poorly differentiated (3), and undifferentiated (4). Metastasis classifications included: bone-only (1), brain-only (2), and bone and brain (3). The variables are further described in Table 1.

### 2.3. Study Outcome

Lung cancer-specific survival was used as the endpoint. Cause-specific survival was calculated to be the difference between the date of diagnosis and death attributed to lung cancer (cause). Follow-up was administratively censored at three and five years from admission to death due to lung cancer.

### 2.4. Statistical Analysis

#### 2.4.1. Model Development

All available data from individual cohorts meeting inclusion criteria were merged, and the resulting dataset was randomly divided into 70:30 training and validation datasets. This allowed for sufficient model training and a large validation set to evaluate model performance within treatment subgroups. Descriptive statistics were used to concisely summarize the distribution of all variables included in the study. The descriptive statistics were utilized to describe the baseline characteristics of patients. Mean and median values were assessed for continuous variables, while frequency distributions were examined for categorical variables. The least absolute shrinkage and selection operator (LASSO) regression was employed to identify feature variables for the models. The Schoenfield residual test was first conducted to assess the hazard proportionality assumption of the variables. Predictive survival models using Cox PH, Weibull AFT, log-normal AFT, and ZBLN AFT statistical approaches were constructed with the training datasets to determine variables’ relative contribution to survival after at least one bone or brain metastatic lung cancer diagnosis. For the selected parametric (AFT) model, the acceleration factor (AF) of the categorical variables was further estimated to assess how much the variables accelerate or decelerate the time to death. AF determination followed these stated criteria: if AF > 1, exposure benefits survival; if AF < 1, exposure is harmful to survival; and if AF = 1, there is no effect from exposure. An AF greater than 1 means that the predictor variable is associated with a longer survival time when compared to the reference variable, and an AF less than 1 means that the predictor variable is associated with a shorter survival time when compared to the reference variable.

#### 2.4.2. Model Performance

The fit of the newly developed prediction model to the dataset was compared with that of two classical AFT models, i.e., the log-normal and Weibull AFT models, as well as the traditional Cox proportional hazard (Cox PH) model, by a rigorous process using the Akaike Information Criterion (AIC) and Bayesian Information Criterion (BIC), whereby the model with the lowest values is considered the best fit.

#### 2.4.3. Model Validation

Internal validation refers to confirming or verifying a theory’s accuracy, reliability, or validity of certain phenomena. The discriminatory ability of the models was estimated using the concordance index (C-index) and calibration plots. The C-index value ranges from 0.50 to 1.00 and shows a positive correlation with the predictive accuracy of the AFT model. It illustrates that the model is accompanied by perfect performance when the value is 1.00. Calibration plots were made using the actual and predicted survival probability to evaluate the predictive performance of the established predictive model. Observation of points close to the diagonal line at a 45° angle indicates ideal calibration. Any variation seen above or below this line may be interpreted as an indication of either an overestimation or an underestimation of survival probability. All statistical analyses above were performed using R version 4.3.1 software (The R Foundation for Statistical Computing, Vienna, Austria; www.r-project.org (accessed on 30 October 2023). All tests were performed with a *p*-value of less than 0.05 being considered statistically significant.

## 3. Results

### 3.1. Description of the Study Cohorts

The baseline characteristics of lung cancer patients with at least one bone or brain metastases are presented in Table 2. A total of 20,412 patients were included in the study. The median survival time for death from lung cancer is 5 (2, 12) months. The average age of the patients at admission is 66 ± 24 years. Most of the patients are White (80%) and had a tumor located in the upper lobe of the lung (53%). In terms of tumor grade, most patients had poorly differentiated (63%) ones and had bimodal therapy (60%). The study cohort was split into the training cohort (*n* = 14,290 patients) and the validation cohort (*n* = 6284 patients). Equal percentages of responses for each variable indicate equal representation in the total, training, and test cohorts.

### 3.2. Proportional Hazard Test

Table 3 shows the result of the evaluation of the proportional hazard assumption of the variables. It reveals that the variables age (*p* < 0.001), race (*p* < 0.002), tumor primary site (*p* < 0.002), grade (*p* < 0.001), TISP (*p* < 0.031), metastases (*p* < 0.001), and treatment modality (*p* < 0.001) did not fulfill the assumption, suggesting that the traditional Cox model will not accurately predict results or will provide misleading statistical significance. As an alternative, the AFT approach was considered.

### 3.3. Baseline Distribution Selection

**Figure 1:**
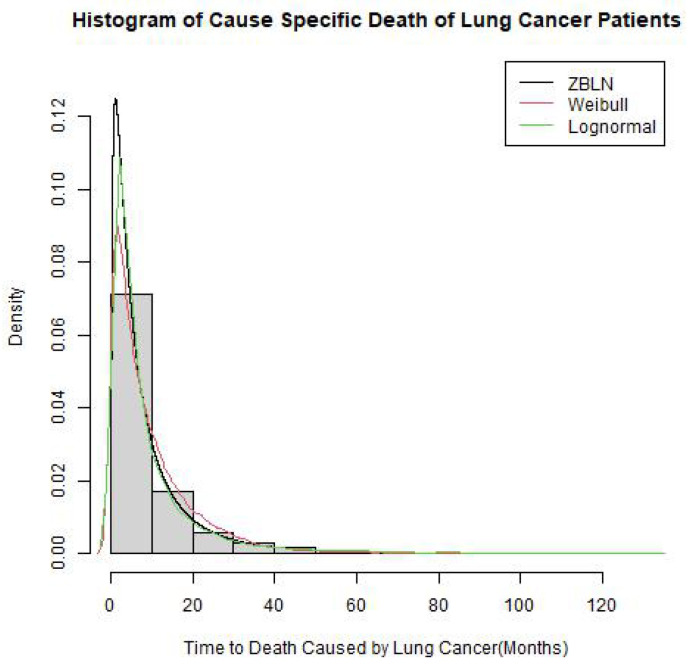
Fitted Distribution of the probability distributions on the CSLCD.

As an alternative to the Cox model, the AFT model approach was considered. The primary assumption for any baseline distribution for the AFT model is that the survival time follows the probability distribution. For this study, three probability distributions were considered as the baseline for the AFT model for the cause-specific survival of the CSLCD patients, namely, the Weibull, log-normal, and ZBLN. Based on the lowest values of AIC (91,578) and BIC (91,568), ZBLN provided the best fit for the survival time compared to the log-normal and Weibull distributions, as shown in Table 4. A calibration plot to show the distribution fits for the survival time is further demonstrated in Figure 1.

### 3.4. Survival Model Development

The ZBLN AFT model, along with the Weibull AFT, log-normal AFT, and Cox PH models, was developed for the data on 3- and 5-year cause-specific survival of lung cancer patients with at least one bone or brain metastasis considering the covariates. The superiority in the goodness of fit of the ZBLN AFT model compared to the other models is shown in Table 5, which reveals that the ZBLN gave the best model fit with the lowest values of AIC (3-year = 63,322 and 5-year = 79,390) and BIC (3-year = 63,495 and 5-year = 79,396), closely followed by the log-normal AFT model (AIC = (3-year = 63,483 and 5-year = 79,632), BIC = (3-year = 63,665 and 5-year = 79,636), then the Weibull AFT (AIC = (3-year = 64,854 and 5-year = 83,066), BIC = (3-year = 65,035 and 5-year = 83,070), and finally Cox PH (AIC = (3-year = 158,691 and 5-year = 211,125), BIC = (3-year = 158,848 and 5-year = 211,287)).

### 3.5. Model Validation

**Figure 2:**
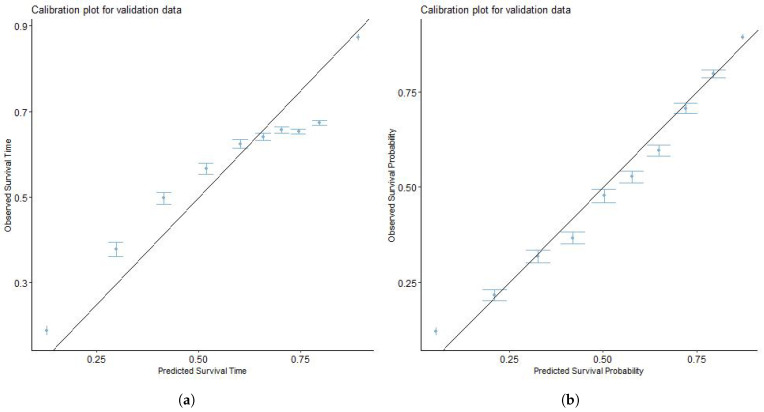
Calibration plots to compare the predictability of Cox PH and ZBLN AFT model on 3-year cause-specific survival of lung cancer patients with at least one bone or brain metastasis. (**a**) Calibration plot of Cox PH model for 3-year cause-specific survival. (**b**) Calibration plot of ZBLN AFT model for 3-year cause-specific survival.

**Figure 3:**
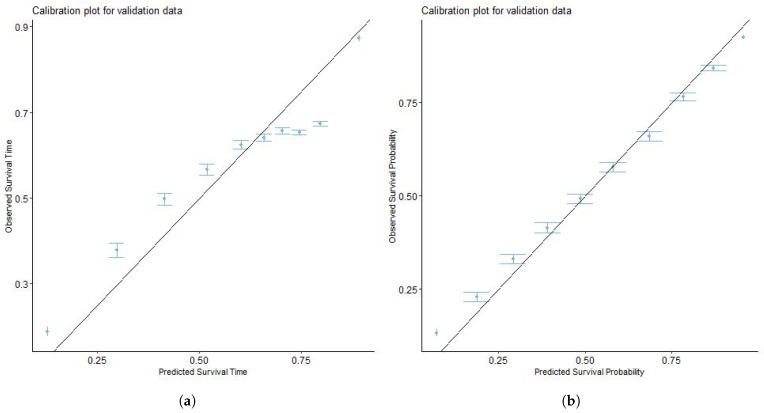
Calibration plots to compare the predictability of Cox PH and ZBLN AFT model on 5-year cause-specific survival of lung cancer patients with at least one bone or brain metastasis. (**a**) Calibration plot of Cox PH model for 5-year cause-specific survival. (**b**) Calibration plot of ZBLN AFT model for 5-year cause-specific survival.

The model obtained from the training cohort was validated using the validation set. For validation, the ZBLN model performed better in discrimination ability (3-year C-index = 0.682, 5-year C-index = 0.667) than the Cox model (3-year C-index = 0.669, 5-year C-index = 0.643). To further establish the superiority of the model in terms of prediction accuracy, the ZBLN model has the lowest RMSE (3-year RMSE = 0.425, 5-year RMSE = 0.667) among the AFT models, indicating better predictive accuracy in survival times. This suggests that the ZBLN model best predicts which individuals will survive longer than others in the validation set. Figure 2 and Figure 3 show the calibration plots of the actual and predicted survival probability using the ZBLN AFT and Cox PH models for 3-year and 5-year CSLCD. The plots show that the ZBLN AFT model gives a better calibration for the 3-year and 5-year CSLCD, as the points are close to the diagonal line.

Based on the results in Table 5 and Table 6, the results of the prediction models using the ZBLN AFT for the 3-year and 5-year CSLCD are presented in Table 7. Table 7 shows that all factors are significant for 3-year CSLCD, while all factors except race are significant for 5-year CSLCD. Furthermore, there is a significant difference in the survival time between reference variables and their respective subcategories, except for race (American Indian vs. White (*p* = 0.781), American Indian vs. Black (*p* = 0.890)), histology (epithelial neoplasm NOS vs. adenomas and adenocarcinoma (*p* = 0.620)), primary site (main bronchus vs. lower lobe (*p* = 0.833), main bronchus vs. overlapping lesion of the lung (*p* = 0.680)), and metastases (bone-only vs. brain-only (*p* = 0.805)) at 3-year CSLCD. In comparison, there is a significant difference in the survival time between reference variables and their respective subcategories for all variables in 5-year CSLCD. A negative sign on the estimate indicates variables that accelerate the patient’s time-to-death compared to the reference variable. In contrast, the variables with positive covariate estimates favor survival (AF > 1). Therefore, this implies that the significant subcategory with the lowest value experiences death faster, and vice versa.

## 4. Discussion

In this section, we discuss the results of the study from both the statistical and clinical perspectives.

This study developed a new accelerated failure time prognostic prediction model using the ZBLN distribution as a baseline. Subsequently, it was validated for 3-year and 5-year CSLCD patients with at least one bone or brain metastasis. The model included factors such as age at diagnosis, tumor grade, primary tumor site, treatment modality, histology, gender, race, and total in situ patient tumors. All predictors are significant for the time to CSLCD at a 0.05 significance level. The ZBLN AFT model demonstrated superior fit compared to Weibull, log-normal AFT, and Cox PH models, evidenced by lower AIC and BIC values (3-year: 63,322, 63,495; 5-year: 79,390, 79,396). Using the concordance index, internal validation revealed the ZBLN AFT model’s highest performance (C-index: 0.682, 0.667), surpassing log-normal, Weibull, and Cox PH models. Evaluation of mean squared error (MSE) highlighted ZBLN’s superior predictive accuracy (MSE: 0.425, 2.628), outperforming log-normal and Weibull models. The calibration plot further confirmed the novel model’s enhanced predictability compared to the Cox PH model. Furthermore, The variables included in the model have a strong association with predictive outcomes, possess attributes that facilitate straightforward measurement, and are often used in routine assessments.

Selecting a suitable survival analysis model is crucial in predictive modeling to precisely predict time-to-event outcomes, particularly in clinical contexts and reliability engineering. The Cox proportional hazard (PH) model is frequently used. However, a more flexible option is to apply generalized parametric models [20,21,22,23,24]. A particular model called ZBLN is highly regarded as the preferred option due to its flexibility to real life data. Generalized parametric models, such as the ZBLN model, provide the ability to choose the distribution that most accurately matches the properties of survival data. Adaptability is essential, as real-world survival times might vary considerably. The benefit of using a generalized method is seen in its ability to customize the model to individual variables, resulting in a better fit to the underlying survival distribution. By selecting an appropriate generalized model, such as ZBLN, that accurately represents the characteristics of the data, researchers can improve the precision of predictions. This makes it a powerful tool for applications that require accurate time-to-event outcomes.

A typical secondary location in patients with lung cancer is in bone and brain metastases. A key objective of the study was to compare the survival of patients with bone metastases alone and those with bone and brain metastases. A striking piece of evidence from this current study is that patients with bone metastases from lung cancer have a higher chance (1.377 times) of living compared to patients with a combination of bone and brain metastases from primary lung cancer. This is not unlikely, as the comorbidity of two major body organs will be weightier than singular morbidity. This study was able to establish an association between age and lung cancer. It revealed that, as the respondents age, the likelihood of dying from lung cancer increases. A study on age as a heterogeneous factor for small-cell lung cancer patients revealed that the probability of metastasis in the elderly was nearly double that in much younger patients [25]. The reasons for the decreased probability of death among the younger age group include their ability to better withstand the stress of treatment and their early response to treatment compared to the elderly. Various other studies have affirmed that lung cancer and its metastases are common among the elderly compared to the younger population [26,27,28,29,30]. Lung cancer death is more likely among the male gender compared to the female gender. The probable reason for this could be the higher incidence of tobacco consumption among men compared to their female counterparts. In a study of sex-specific trends in lung cancer incidence and survival, it was found that, although a higher proportion of women were diagnosed with the disease, the probability of dying was significantly higher in men at all stages. Another likely reason for this may be men’s poor health-seeking behavior compared to women. Previous studies on lung cancer have demonstrated that Whites survive lung cancer better than Blacks, and the major reason adduced to this is because Blacks are usually diagnosed at a more advanced stage. Further studies that controlled for certain factors like the stage of the disease, treatment received, and socioeconomic factors, however, showed no difference in the survival between the two races [29]. In addition, Jones and his colleagues also studied the effect of African ancestry and found that it was also not associated with mortality or survival in lung cancer [28]. Although this study found a statistical difference in race, the effect of socioeconomic factors was, however, not considered because the variable was not part of the data. Males may end up presenting late to the hospital, which can worsen the prognosis of the disease [31]. Patients who have had two or more treatment modalities are three times more likely to survive lung cancer than patients not treated or with only one treatment measure. As is known, prevention is better than cure. Prevention could be primary, secondary, or tertiary. Early detection and prompt treatment, the secondary form of prevention, are highly recommended in the prevention/treatment of lung cancer. On the contrary, tertiary prevention focuses on limiting disability or complication. Metastases in patients with lung cancer are a form of difficulty that could have been prevented if detected early and if treatment is started immediately. In this study, the histological type of lung cancer was also statistically significant with the probability of developing metastasis. The primary histological type associated with metastasis progression was squamous cell carcinoma. This is quite different from the findings of another study where adenocarcinoma was the most prevalent histological type of lung cancer associated with the progression of metastases among men and women [30]. The result of this current study is also in tandem with a similar study done at University College Hospital, where squamous cell carcinoma was found to be more common [32]. There is a strong link between types of metastases in patients and the duration of their survival. In this current study, patients with bone and brain metastases were significantly survival time-dependent when studied for three years, whereas those patients with brain metastases were not survival time-dependent. This is quite different for both groups when studied for five years. This means that types of metastases or multiple metastases determine the length of survival when analyzed for a longer time. The longer survival duration is closely linked to the types and numbers of metastases to other body parts.

## 5. Limitation of the Study

The primary limitation of the SEER database is its deficiency in providing data per patient about smoking habits and other socioeconomic characteristics that can contribute to lung cancer survival. The presence of this constraint poses significant difficulty in disentangling the possible confounding influences of gender, smoking behaviors, and socioeconomic standing. Secondly, the database contains a lot of missing data, which resulted in high exclusions from the final dataset used in the study. The present investigation uses the Surveillance, Epidemiology, and End Results (SEER) database; therefore, it warrants caution in generalizing the findings to patients outside the United States. Consequently, it would be essential to have this model externally validated using cohorts of data from outside the U.S.

## 6. Further Research

This study is limited to cause-specific survival of lung cancer with at least one bone or brain metastasis. Further research can be done to assess the overall survival, considering an accelerated failure time competing risk model. This will involve those who died due to lung cancer and other related causes.

## 7. Conclusions

Our work successfully introduced an innovative accelerated failure time model to predict cause-specific survival in lung cancer patients. The model exhibited strong predictive performance, showcasing high precision and predictive capability. These findings have important significance for clinicians, providing a powerful tool to assess the survival of patients with lung cancer who present with one or a combination of bone and brain metastases. The model’s effectiveness creates chances to implement tailored treatment approaches, improving the quality of patient care and results. Moreover, our research highlights the crucial significance of using generalized parametric models, such as the ZBLN model, a generalized version of the log-normal distribution, in predictive modeling for time-to-event outcomes. The ZBLN model’s versatility, accuracy, and ability to capture the complexities of survival data make it a powerful tool in applications requiring precise predictions, especially in clinical domains, compared to the commonly used Cox PH model. Our study demonstrates that using advanced modeling tools improves predicted accuracy and our comprehension of the intricate dynamics of survival analysis. This research encourages the adoption of generalized parametric models for their ability to provide nuanced insights and improve the precision of predictions, ultimately contributing to advancements in clinical decision-making.

## Figures and Tables

**Table 1:** Data variable descriptions.

Variable	Description	Data Type	Data Format
CSLCD	Time to death caused by lung Ccancer	Continuous	1,2,3,4,5,…
Age	Age of patients	Continuous	1,2,3,4,5,…
Race	Race of patients	Categorical	1,2,3,4
Histology	Histology	Categorical	1,2,3
Treatment Modality	Treatments administered	Categorical	1,2,3,4
TISD	In situ malignant tumors	Continuous	1,2,3,4,5,…
Histological Grade	Histological grade of tumor	Categorical	1,2,3,4
Gender	Gender of the patients	Categorical	1,2
Primary Site	Tumor primary location	Categorical	1,2,3,4,5,6
Censor	Censored value	Categorical	0,1
Metastases	Metastases	Categorical	1,2,3

**Table 2:** Descriptive statistics of patients’ characteristics.

Variable	Total Cohort	Training Cohort	Validation Cohort
	Percentages	Percentages	Percentages
Survival Time in Months	5 (2, 12) *	5 (2, 12) *	5 (2, 12) *
Uncensored (3-year)	13,298 (65%)	9331 (65%)	3967 (65%)
Uncensored (5-year)	17,753 (87%)	12,441 (87%)	5312 (87%)
Age of Patients	66 (11) **	66 (11) **	67 (10) **
Race			
American Indian	108 (0.5%)	76 (0.5%)	32 (0.5%)
Asian	1614 (7.9%)	1148 (8.0%)	466 (7.6%)
Black	2274 (11%)	1608 (11%)	666 (11%)
White	16,416 (80%)	11,458 (80%)	4958 (81%)
Sex			
Male	11,447 (56%)	8042 (56%)	3405 (56%)
Female	8965 (44%)	6248 (44%)	2717 (44%)
Primary Site			
Main Bronchus	993 (4.9%)	699 (4.9%)	294 (4.8%)
Upper Lobe	10,913 (53%)	7610 (53%)	3303 (54%)
Middle Lobe	863 (4.2%)	571 (4.0%)	292 (4.8%)
Lower Lobe	5370 (26%)	3828 (27%)	1542 (25%)
Lung NOS	214 (1.0%)	145 (1.0%)	69 (1.1%)
Overlapping Lesion of Lung	2059 (10%)	1437 (10%)	622 (10%)
Grade			
Well-Differentiated	872 (4.3%)	616 (4.3%)	256 (4.2%)
Moderately Differentiated	4637 (23%)	3284 (23%)	1353 (22%)
Poorly Differentiated	12,927 (63%)	9011 (63%)	3916 (64%)
Undifferentiated	1976 (9.7%)	1379 (9.7%)	597 (9.8%)
TISP	1 (1, 1) *	1 (1, 1) *	1 (1, 1) *
Metastasis Type			
Bone Only	9837 (48%)	6863 (48%)	2974 (49%)
Bone and Brain	3408 (17%)	2410 (17%)	998 (16%)
Brain Only	7167 (35%)	5017 (35%)	2150 (35%)
Treatment Modality			
No Treatment	5640 (28%)	3975 (28%)	1665 (27%)
Monotherapy	862 (4.2%)	602 (4.2%)	260 (4.2%)
Bimodal Therapy	12,146 (60%)	8489 (59%)	3657 (60%)
Trimodal Therapy	1764 (8.6%)	1224 (8.6%)	540 (8.8%)
Histology			
Epithelial Neoplasms	5024 (25%)	3477 (24%)	1547 (25%)
Squamous Cell Neoplasms	3428 (17%)	2400 (17%)	1028 (17%)
Adenomas and Adenocarcinomas	11,188 (55%)	7890 (55%)	3298 (54%)
Others	772 (3.8%)	523 (3.7%)	249 (4.1%)

* Median values (with lower and upper quartiles); ** Mean (with standard deviation).

**Table 3:** Proportional hazard assumption test.

	3-Year	5-Year
Variable	*p*-Value	*p*-Value
Age	<0.001 *	<0.001 *
Race	0.002 *	0.001 *
Gender	0.102	0.003
Tumor Primary Site	0.002 *	0.003
Grade	<0.001 *	<0.001 *
TISP	0.031 *	0.067
Metastases	<0.001 *	0.001
Treatment	<0.001 *	<0.001
Histology	0.087	0.249

* Variables that do not satisfy the proportional hazard assumption

**Table 4:** Comparative results of model fit for probability distributions on the survival time of the training cohort.

Model	-log-likelihood	AIC	BIC
Weibull	46,402	92,808	92,823
Log-normal	45,805	91,630	91,615
ZBLN	45,786	91,578	91,568

**Table 5:** Comparative results of the model fit of the training cohort of the CSLCD using the four survival models.

		3-Year					5-Year	
Model	-log-likelihood	AIC	BIC			-log-likelihood	AIC	BIC
Cox	79,323	158,691	158,848			105,540	211,125	211,287
Weibull	33,305	64,854	65,035			41,531	83,066	83,070
Log-normal	31,717	63,483	63,665			39,814	79,632	79,636
ZBLN	31,658	63,322	63,495			39,692	79,390	79,396

**Table 6:** Comparative results of the predictive ability of the four survival models on the validation cohort of the CSLCD.

	3-Year				5-Year	
Model	RMSE	C-Index			RMSE	C-Index
Cox	- *	0.669			- *	0.643
Weibull	7.344	0.636			6.264	0.652
Log-normal	0.453	0.665			5.739	0.666
ZBLN	0.425	0.682			2.628	0.667

* Not applicable to the Cox PH model.

**Table 7:** Multivariate analysis of the 3- and 5-year cause-specific survival of lung cancer patients with at least one bone or brain metastasis using the ZBLN AFT model.

		3-Year								5-Year	
Variable	Estimates	AF	*p*						Estimates	AF	*p*
**Age**	−0.003	0.997	0.025						−0.007	0.993	0.000
**Race**											
American Indian (Ref)	-	-	-						-	-	-
Asian	0.414	1.513	0.015						0.263	1.300	0.053
Black	−0.047	0.954	0.781						−0.062	0.940	0.646
White	0.023	1.023	0.890						−0.025	0.975	0.847
**Gender**											
Male (Ref)	-	-	-						-	-	-
Female	0.066	1.069	0.005						0.137	1.146	<0.001
**Treatment Modality**											
No Treatment (Ref)	-	-	-						-	-	-
Monotherapy	−0.347	0.707	0.000						−0.117	0.889	0.022
Bimodal Therapy	1.238	3.448	0.000						1.053	2.867	0.000
Trimodal Therapy	1.237	3.447	0.000						1.109	3.032	0.000
**Histology**											
Epithelial Neoplasms, NOS	-	-	-						-	-	-
Squamous Cell Neoplasms	−0.132	0.876	0.001						−0.297	0.742	0.000
Adenomas and Adenocarcinomas	0.016	1.016	0.620						−0.083	0.920	0.002
Others	−0.355	0.701	0.000						−0.162	0.851	0.004
**Primary Site**											
Main Bronchus (Ref)	-	-	-						-	-	-
Upper Lobe	0.183	1.201	0.001						−0.197	0.821	0.002
Middle Lobe	−0.706	0.494	0.000						−0.145	0.864	0.002
Lower Lobe	−0.012	0.988	0.833						0.215	1.240	0.000
Lung NOS	−0.823	0.439	0.000						−1.175	0.308	0.000
Overlapping Lesion of the Lung	−0.027	0.974	0.680						−0.456	0.633	0.000
**Grade**											
Well-Differentiated (Ref)	-	-	-						-	-	-
Moderately Differentiated	0.164	1.179	0.008						0.141	1.152	0.005
Poorly Differentiated	−0.122	0.885	0.038						−0.219	0.803	0.000
Undifferentiated	−0.345	0.708	0.000						−0.440	0.643	<0.001
**TISP**	0.202	1.223	0.000						0.161	1.174	0.00
**Metastases**											
Bone Only (Ref)	-	-	-						-	-	-
Bone and Brain	−0.320	0.726	0.000						−0.305	0.736	0.000
Brain Only	0.007	1.007	0.805						−0.079	0.924	0.000

AF = Accelerator Factor.

## Data Availability

The data were abstracted and readily available from the Surveillance, Epidemiology, and End Results (SEER) database (https://seer.cancer.gov accessed on 30 October 2023).
